# Evaluate the Efficacy of the Axl inhibitor Bemcentinib in the 5xFAD mouse model of Alzheimer’s Disease

**DOI:** 10.1002/alz.089525

**Published:** 2025-01-03

**Authors:** Guyin Chen, Gizem Ozturk, Sílvia Porta, Virginia M.‐Y. Lee

**Affiliations:** ^1^ Center for Neurodegenerative Disease Research, Department of Pathology and Laboratory Medicine, University of Pennsylvania, Perelman School of Medicine, Philadelphia, PA USA; ^2^ Institute on Aging, University of Pennsylvania, Perelman School of Medicine, Philadelphia, PA USA

## Abstract

**Background:**

Alzheimer’s disease (AD) is a neurodegenerative disease characterized by the presence of phosphorylated tau neurofibrillary tangles and extracellular deposits of amyloid beta plaques (Aβ) in the brain. Microglia cells have been proposed to be involved in amyloid plaque formation since activated microglia produce inflammatory cytokines that contribute to a hostile neuronal environment, exacerbating AD pathogenesis.

**Method:**

We aim to evaluate if the pharmacological inhibition of the myeloid/microglial receptor tyrosine kinase AXL, with bemcentinib (BGB) could be used as a novel therapeutic approach for AD. The objective is to evaluate the efficacy of the BGB inhibiting Axl activity in the brain of the 5xFAD mouse model, which recapitulates pathological hallmarks of AD, including Aβ plaques formation and overactive microglia. We evaluated target engagement and performed an efficacy study in 5xFAD and WT mice orally dosed with BGB and Vehicle (Veh) as control. Mice were dosed twice a day for 1 week. Mice were anesthetized to collect CSF and plasma and perfused to obtain their brains.

**Result:**

In the present work, we have used the pharmacological Axl inhibitor BGB, to evaluate the therapeutic potential of targeting Axl signaling in 5xFAD mice. We have evaluated the effect of Axl inhibition measuring changes in total Axl levels and its soluble fragment (sAxl) across the hippocampus, plasma, and CSF of 5xFAD mice and WT mice. As expected, we found a significant upregulation of Aβ plaques and microglia in 5xFAD mice, and interestingly, a concomitant upregulation of Axl levels in the hippocampus of 5xFAD mice. We observed, although not statistically significant, a trend of increasing levels of sAxl in CSF and plasma of mice receiving BGB. Unfortunately, no significant effect of BGB drug was detected in 5xFAD mice when Aβ‐plaque burden and Iba1 immunoreactivity was measured after the treatment.

**Conclusion:**

Overall, the preliminary findings do not show a significant effect of Axl inhibitor BGB in the brain. The reduced BBB penetrance of the drug could be a potential explanation for lack of significant changes in Axl levels and microglia activation. Further studies of increasing dosing time and cohort size could potentially improve the outcome.

